# *ADRB2* Arg16Gly Polymorphism, Lung Function, and Mortality: Results from the Atherosclerosis Risk in Communities Study

**DOI:** 10.1371/journal.pone.0000289

**Published:** 2007-03-14

**Authors:** Jill M. Ferdinands, David M. Mannino, Marta L. Gwinn, Molly S. Bray

**Affiliations:** 1 Air Pollution and Respiratory Health Branch, Division of Environmental Hazards and Health Effects, National Center for Environmental Health, Centers for Disease Control and Prevention, Atlanta, Georgia, United States of America; 2 Division of Pulmonary, Critical Care, and Sleep Medicine, University of Kentucky College of Medicine, Lexington, Kentucky, United States of America; 3 National Office of Public Health Genomics, Centers for Disease Control and Prevention, Atlanta, Georgia, United States of America; 4 Department of Pediatrics, Baylor College of Medicine, Houston, Texas, United States of America; Innsbruck Medical University, Austria

## Abstract

**Background:**

Growing evidence suggests that the Arg16Arg genotype of the beta-2 adrenergic receptor gene may be associated with adverse effects of beta-agonist therapy. We sought to examine the association of beta-agonist use and the Arg16Gly polymorphism with lung function and mortality among participants in the Atherosclerosis Risk in Communities study.

**Methodology and Principal Findings:**

We genotyped study participants and analyzed the association of the Arg16Gly polymorphism and beta-agonist use with lung function at baseline and clinical examination three years later and with all-cause mortality during 10 years of follow-up. Lung function was characterized by percent-predicted forced expiratory volume in 1 second. Associations were examined separately for blacks and whites. Black beta-agonist users with the Arg/Arg genotype had better lung function at baseline and at the second clinical visit than those with Arg/Gly and Gly/Gly genotypes. Adjusted mean percent-predicted FEV_1_ was 21% higher in Arg/Arg subjects compared to Gly/Gly at baseline (p = 0.01) and 20% higher than Gly/Gly at visit 2 (p = 0.01). Arg/Gly subjects had adjusted percent-predicted FEV_1_ 17% lower than Arg/Arg at baseline but were similar to Arg/Arg subjects at visit 2. Although black beta-agonist users with the Arg/Arg genotype appeared to have better crude survival rates, the association between genotype and all-cause mortality was inconclusive. We found no difference in lung function or mortality by genotype among blacks who did not use beta-agonists or among whites, regardless of beta-agonist use.

**Conclusions:**

Black beta-agonist users with the *ADRB2* Arg16Arg genotype had better lung function, and, possibly, better overall survival compared to black beta-agonist users with the Gly16Gly genotype. Our findings highlight the need for additional studies of sufficient size and statistical power to allow examination of outcomes among beta-agonist users of different races and genotypes.

## Introduction

Asthma is an important cause of morbidity and mortality in the United States, where African-Americans bear a disproportionate burden of asthma hospitalization and have asthma mortality rates 3 to 4 times higher than whites[1]. The reasons for this disparity are unclear but are thought to be related to poorer access to health care, living in an urban environment, or other unknown factors [2].

Beta-agonist medications are a mainstay of asthma treatment and are clearly important for symptom control. Some studies, however, have implicated heavy beta-agonist use in fatal asthma exacerbations, although this association remains controversial [3]–[5]. In 2003, a large clinical trial of the long-acting beta-agonist salmeterol was halted after a greater-than-expected number of asthma fatalities in the treatment arm; this increase in mortality was statistically significant among black but not white study participants [6].

Agonist-induced activation of the ß_2_-adrenergic receptor (ß_2_AR) causes the dilatation of airway smooth muscle responsible for the therapeutic effect of beta-agonist medications[7]. The Arg16Gly polymorphism of the ß_2_AR gene (*ADRB2*) is a genetic variant associated with receptor down-regulation, and growing evidence suggests that it plays an important role in beta-agonist response [8], [9]. Recent studies, including a randomized clinical trial, indicate that asthma patients with the Arg/Arg genotype may experience adverse effects from beta-agonist use, including deterioration of lung function [10]–[16], and recent literature reviews suggest an emerging consensus that the Arg16 allele is associated with clinically significant, unsatisfactory response to beta-agonists[17]–[21]. Frequency of the Arg16 allele is about 39% to 42% in whites and about 49% in blacks [22], [23].

We sought to describe the association of beta-agonist use and the *ADRB2* Arg16Gly polymorphism with lung function and mortality among participants in the Atherosclerosis Risk in Communities (ARIC) study [24], and to examine if these associations vary by race. Our analysis considered factors for which data were available from the ARIC database, including smoking history, asthma history, respiratory symptoms, body mass index (BMI), and use of other respiratory medications. To our knowledge, this is the first large, population-based study to examine the association between *ADRB2* Arg16Gly polymorphism, lung function, beta-agonist use, and mortality.

## Methods

### Subjects

The ARIC cohort consists of 15,792 adults selected by probability sampling from four US communities [24]. At baseline clinical examination in 1987–1989 and follow-up examination 3 years later (“visit 2”), participants underwent pulmonary function testing and provided information on respiratory symptoms and diagnoses, BMI, smoking history, and medication use. Follow-up through 1998 was available for this analysis. Analysis was limited to black and white ARIC participants who had baseline demographic data, pulmonary function testing, smoking history, and *ADRB2* Arg16Gly genotype.

### Variable Definition

Subjects were classified as beta-agonist users at baseline or visit 2 if they reported use of inhaled beta-agonists in the 2 weeks prior to the baseline exam or 2 weeks prior to the clinical follow-up exam, respectively. Subjects were classified as users of other respiratory medications if they reported use of inhaled corticosteroids, theophylline, oral steroids, or other respiratory medications in the same time frames.

Measures of lung function were forced expiratory volume in 1 second (FEV_1_) and FEV_1_/FVC ratio. Spirometry was conducted using a volume displacement, water-sealed spirometer. At least three acceptable spirograms were obtained from a minimum of five forced expirations. The best single spirogram was identified by computer and confirmed by a technician. Quality assurance was provided by the ARIC Pulmonary Function Center, and the procedures followed contemporary American Thoracic Society guidelines. We used prediction equations derived from NHANES to estimate percent-predicted FEV_1_ [25]. As detailed in the on-line supplement (Text S1), subjects were assigned a baseline respiratory symptom score of 0 to 9 based on number of affirmative responses to 9 respiratory symptom questions. Severity of obstructive lung disease was classified per criteria of the Global Initiative for Chronic Obstructive Lung Disease (GOLD) [26] and collapsed into an indicator variable defined as GOLD Stage II or more severe (equivalent to percent-predicted FEV_1_<80% and FEV_1_/FVC<70%).

Respondents with affirmative responses to “Have you ever smoked cigarettes?” and “Do you now smoke cigarettes?” were classified as “ever smokers” and “current smokers,” respectively. BMI was calculated as weight divided by height squared (kg/m^2^) [27]. Education level was less than high school, completion of high school, or more than high school.

### Genotyping

Genotyping of the *ADRB2* Arg16Gly polymorphism was performed using the TaqMan assay (Applied Biosystems, Foster City, Calif.). A 121-bp product was amplified using 0.9 µM each of the forward primer 5′-CGGCAGCGCCTTCTTG-3′ and the reverse primer 5′-GGCCAGGACGATGAGAGACA-3′, 50 ng DNA, 5.0 mM MgCl_2_, and 1×TaqMan Universal PCR Master Mix containing AmpliTaq Gold DNA Polymerase in a 22-µl reaction volume. After an initial step of 2 minutes at 50°C and 10 minutes at 95°C to activate the AmpliTaq Gold, products were amplified using 40 cycles of 15 seconds at 95°C and 1 minute at 62°C. A total of 0.2 µM of each of the sequence-specific probes 5′-6FAM-CTGGCACCCAATAGAAGCCATGC-TAMRA-3′ and 5′-VIC-CTGGC ACCCAATGGAAGCCATG-TAMRA-3′ was used in the allele discrimination assay. Allele detection and genotype calling were performed using the ABI 7900 and the Sequence Detection System software (Applied Biosystems).

### Analysis

Cross-sectional univariate and multivariate linear regression were used to examine the association between lung function and genotype, stratified by beta-agonist use and race and controlling for age, sex, smoking status, respiratory symptom score, and use of other respiratory medications.

Crude differences in survival by genotype were examined with the Kaplan-Meier estimator stratified by race and beta-agonist use, tested with the log-rank statistic. Cox proportional hazards models stratified by race and beta-agonist use were used to estimate hazard ratios by genotype, controlling for age, sex, FEV_1_/FVC ratio, respiratory symptom score, and smoking status. Analyses were conducted with SASv9 (Cary, NC).

## Results

Of 15,552 eligible subjects, 567 (3.4%) were excluded because they did not consent to genotyping or genotyping was unsuccessful. Of the remaining subjects, 46 lacked pulmonary function data and 5 lacked smoking history, leaving 14,934 subjects for analysis. Of these, 4016 (27%) were black. Subject characteristics are summarized in Table 1. Subjects had a mean age of 54 years at baseline and 55% were female. Overall, 1.7%, 2.1%, and 1% of subjects reported beta-agonist use at baseline, visit 2, and both visits, respectively. Black subjects were slightly younger, had better baseline lung function, and were less likely to suffer from obstructive lung disease; in addition, they were more likely to be female and never smokers (p<0.01 for all).

**Table 1:** Demographic, phenotypic, and genotypic characteristics by race*

	Black	White	All
	Subjects	Subjects	Subjects
	n = 4016	n = 10,918	n = 14,934
**Age in years, mean±SD** †	53.5±5.8	54.4±5.7	54.1±5.8
**Sex, n (%)** †			
Male	1534 (38)	5163 (47)	6697 (45)
Female	2482 (62)	5755 (53)	8237 (55)
**Cigarette smoking, n (%)** †			
Current	1259 (32)	2995 (27)	4254 (28)
Former	952 (24)	3857 (35)	4809 (32)
Never	1801 (45)	4060 (37)	5861 (39)
**Percent-predicted FEV_1_, mean±SD** †	95.6±17.8	92.6±16.7	93.4±17.1
**FEV_1_/FVC ratio, mean±SD** †	0.76±0.08	0.74±0.08	0.74±0.08
**Respiratory symptom score (0–9)** †			
Mean	0.8	1.0	0.9
Median (interquartile range)	0 (0–1)	0 (0–1)	0 (0–1)
***ADRB2*** ** Arg16Gly genotype, n (%)** †			
Arginine/Arginine	971 (24)	1449 (13)	2420 (16)
Arginine/Glycine	1977 (49)	5204 (48)	7181 (48)
Glycine/Glycine	1068 (27)	4265 (39)	5333 (36)
**Beta-agonist use, n (%)**			
At baseline (visit 1)	63 (1.6)	189 (1.7)	252 (1.7)
At clinical follow-up exam (visit 2)	69 (1.7)	245 (2.2)	314 (2.1)
At visits 1 and 2	33 (0.8)	123 (1.1)	156 (1.0)
**Obstructive lung disease at baseline (GOLD Stage II or worse)** ** †			
**Yes**	658 (16)	2657 (24)	3315 (22)
**No**	3354 (84)	8258 (76)	11612 (78)

* FEV_1_ denotes forced expiratory volume in 1 second, FVC forced vital capacity, SD standard deviation, *ADRB2* beta_2_ adrenergic receptor gene

** GOLD Stage II obstructive lung disease defined as percent-predicted FEV_1_<80% and FEV_1_/FVC<70% based on criteria from the Global Initiative for Chronic Obstructive Lung Disease [26]

† p<0.05 for test of difference across race using χ^2^ test of heterogeneity for *R*x2 contingency table for categorical variables or ANOVA *F* test for continuous variables, transformed as needed

*ADRB2* Arg16Gly genotype frequencies varied by race (p<0.01), with 49% Arg/Gly, 27% Gly/Gly, and 24% Arg/Arg among blacks and 48% Arg/Gly, 39% Gly/Gly, and 13% Arg/Arg among whites. Arg16 allele frequencies were 0.49 and 0.37 among blacks and whites, respectively, and were consistent with Hardy-Weinberg equilibrium within each race. Overall, genotype frequencies did not differ by age or sex, and genotype was not associated with obstructive lung disease. Subjects with the Arg/Arg genotype were more likely to be never smokers (p = 0.01).

Characteristics of subjects reporting beta agonist use at baseline are provided in Table 2. Among black beta-agonist users, percent-predicted FEV_1_ and respiratory symptom score varied by genotype, with Arg/Arg subjects having better lung function and fewer symptoms, as described below (p = 0.01 and p = 0.02, respectively). Among white beta-agonist users, only smoking status varied by genotype, with Arg/Arg subjects more likely to be never smokers (p = 0.03). Black beta-agonist users with Arg/Arg were also more likely to be never smokers but this association was not statistically significant.

**Table 2:** Characteristics of subjects reporting beta-agonist use at baseline by race and ***ADRB2*** **Arg16Gly genotype**

	Black beta-agonist users			White beta-agonist users		
	Arg/Arg	Arg/Gly	Gly/Gly	Arg/Arg	Arg/Gly	Gly/Gly
	n = 11	n = 35	n = 17	n = 27	n = 88	n = 74
Age in years, mean±SD	54.4±4.7	54.3±6.2	55.5±6.0	55.0±5.9	57.1±5.9	56.1±6.0
Sex, n (%)						
Male	2 (18)	12 (34)	4 (24)	10 (37)	49 (56)	34 (46)
Female	9 (82)	23 (66)	13(76)	17 (63)	39 (44)	40 (54)
Cigarette smoking, n (%) †_whites only_						
Current	1 (9)	8 (23)	5 (29)	5 (19)	28 (32)	24 (32)
Former	3 (27)	13 (37)	8 (24)	8 (30)	42 (48)	27 (36)
Never	7 (64)	14 (40)	14 (47)	14 (52)	18 (20)	23 (31)
Percent-predicted FEV_1_, mean±SD †_blacks only_	87±31	66±21	63±15	67±27	63±24	65±27
FEV_1_/FVC ratio mean±SD	0.70±0.14	0.62±0.13	0.61±0.11	0.60±0.14	0.58±0.13	0.59±0.16
Respiratory symptom score (0–9), mean †_blacks only_	3.9	5.2	4.6	5.8	5.1	5.2
Median (IQR)	4 (0–7)	5 (4–6)	5 (3–6)	6 (4–8)	5(3.5–9)	5 (3–8)
Obstructive lung disease at baseline (GOLD Stage> = 2)*						
Yes	5 (45)	26 (74)	13 (76)	22 (81)	75 (85)	53 (72)
No	6 (55)	9 (26)	4 (24)	5 (19)	13 (15)	21 (28)
Inhaled corticosteroid use at baseline, n (%)						
Yes	1 (9)	2 (6)	4 (24)	4 (15)	8 (9)	10 (14)
No	10 (91)	22 (94)	13 (76)	23 (85)	80 (91)	64 (86)

* GOLD Stage II obstructive lung disease defined as percent-predicted FEV_1_<80% and FEV_1_/FVC<70% based on criteria from the Global Initiative for Chronic Obstructive Lung Disease [26]

† p<0.05 for test of difference across genotype using χ^2^ test of heterogeneity for *R*x2 contingency table for categorical variables or ANOVA *F* test for continuous variables, transformed as needed

**Figure 1:**
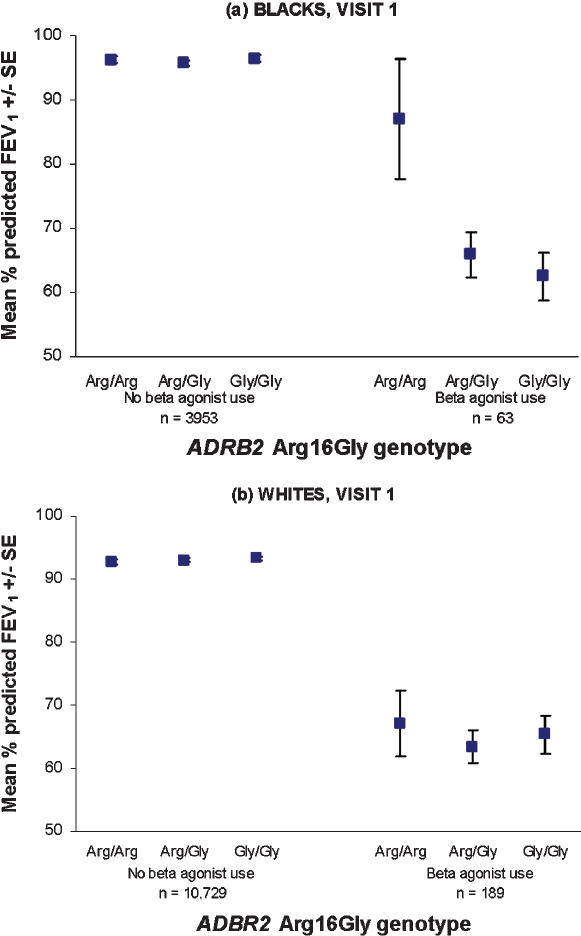
Baseline percent-predicted forced expiratory volume in 1 second (FEV_1_) by *ADRB2* Arg16Gly genotype, stratified by beta-agonist use in blacks (upper panel, p = 0.01) and whites (lower panel, p = 0.77)

Among black subjects with beta-agonist use at baseline (n = 63), those with the Arg/Arg genotype had better baseline lung function than those with Arg/Gly or Gly/Gly genotypes [crude percent-predicted FEV_1_ 87% (95% CI 69% to 105%) for Arg/Arg, 66% (95% CI 60% to 73%) for Arg/Gly, and 63% (95% CI 55% to 70%) for Gly/Gly, p = 0.01 for trend, Fig. 1]. Results were similar in a model controlling for age, sex, smoking status, respiratory symptom score, and use of other respiratory medication, in which adjusted mean percent-predicted FEV_1_ was 21% higher in Arg/Arg subjects compared to Gly/Gly (p = 0.01). We obtained a qualitatively similar result using the endpoint of baseline FEV_1_/FVC ratio, although the relationship was not statistically significant. We found no difference in lung function by genotype among white subjects with beta-agonist use at baseline and among non-users of beta-agonists of either race. Crude and adjusted lung function estimates by race and genotype, stratified by beta-agonist use, are summarized in Table 3.

**Table 3:** Crude and adjusted baseline lung function by race and *ADRB2* Arg16Gly genotype for subjects with (top) and without (bottom) baseline beta-agonist use

	Blacks with baseline beta-agonist use			Whites with baselinebeta-agonist use		
	Arg/Arg	Arg/Gly	Gly/Gly	Arg/Arg	Arg/Gly	Gly/Gly
	n = 11	n = 35	n = 17	n = 27	n = 88	n = 74
Crude percent-predicted FEV_1_, mean (95%CI)	87 (69–105)	66 (60–73)	63 (55–70)	67 (57–77)	63 (58–68)	65 (59–71)
Adjusted percent-predicted FEV_1_, mean (95%CI)	83 (70–97)	67 (60–74)	63 (53–73)	61 (53–69)	66 (61–70)	65 (60–69)
Crude FEV_1_/FVC ratio, mean (95%CI)	0.70 (0.61–0.78)	0.62 (0.58–0.67)	0.61 (0.55–0.66)	0.60 (0.55–0.65)	0.58 (0.55–0.61)	0.59 (0.56–0.63)
Adjusted FEV_1_/FVC ratio, mean (95%CI)	0.69 (0.61–0.76)	0.63 (0.59–0.67)	0.61 (0.55–0.66)	0.57 (0.52–0.61)	0.59 (0.57–0.62)	0.59 (0.56–0.62)

	Blacks without baseline beta-agonist user			Whites without baseline beta-agonist user		
	Arg/Arg	Arg/Gly	Gly/Gly	Arg/Arg	Arg/Gly	Gly/Gly
	n = 960	n = 1942	n = 1051	n = 1422	n = 5116	n = 4191
Crude percent-predicted FEV_1_, mean (95%CI)	96 (95–97)	96 (95–97)	96 (95–98)	93 (92–94)	93 (93–94)	93 (93–94)
Adjusted percent-predicted FEV_1_, mean (95%CI)	96 (95–98)	96 (95–96)	96 (95–97)	93 (92–94)	93 (93–93)	93 (93–94)
Crude FEV_1_/FVC ratio, mean (95%CI)	0.77 (0.76–0.77)	0.77 (0.76–0.77)	0.77 (0.76–0.77)	0.74 (0.74–0.74)	0.74 (0.74–0.74)	0.74 (0.74–0.74)
Adjusted FEV_1_/FVC ratio, mean (95%CI)	0.77 (0.76–0.77)	0.77 (0.76–0.77)	0.77 (0.76–0.77)	0.74 (0.74–0.74)	0.74 (0.74–0.74)	0.74 (0.74–0.74)

* Adjusted for age, sex, baseline smoking status, respiratory symptom score, and use of other respiratory medication

Black beta-agonist users with the Arg/Arg genotype reported slightly fewer respiratory symptoms at baseline than those with Arg/Gly and Gly/Gly (median of 4 symptoms for Arg/Arg versus 5 symptoms for Arg/Gly and Gly/Gly, p = 0.02). Black beta-agonist users with the Gly/Gly genotype were more likely to report taking inhaled corticosteroids at baseline (24% vs 6% for Arg/Gly and 9% for Arg/Arg), although this difference was not statistically significant (p = 0.15).

A similar relationship was observed among black subjects reporting beta-agonist use at visit 2 (n = 69, 62 of whom provided pulmonary function data). Those with the Arg/Arg genotype had percent-predicted FEV_1_ that was, on average, 20% higher than those with the Gly/Gly genotype, controlling for the same variables as above (p = 0.01). However, at visit 2, heterozygotes had lung function levels similar to those with the Arg/Arg genotype. No differences in lung function by genotype were observed in whites reporting beta-agonist use at baseline (n = 189) or at visit 2 (n = 231). Because only 31 black subjects reported beta-agonist use at both visits, there were too few subjects with each genotype to assess temporal variation in pulmonary function by genotype and race.

**Figure 2:**
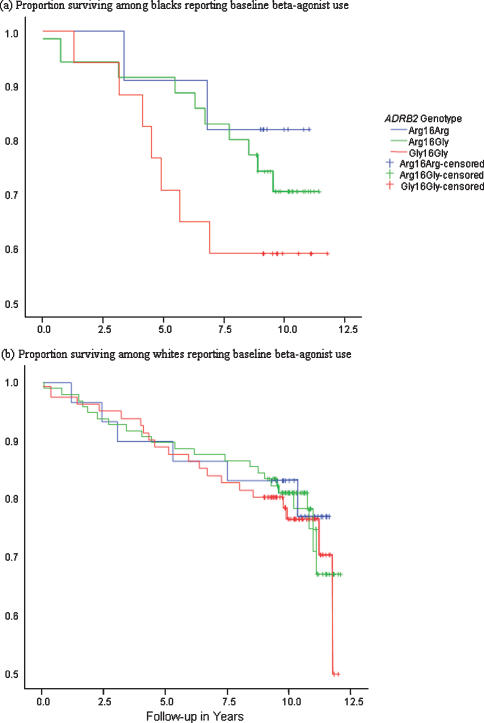
Crude survival among black (upper panel, n = 63, p = 0.33) and white (lower panel, n = 189, p = 0.27) study participants reporting baseline beta-agonist use by *ADRB2* Arg16Gly genotype

Subjects were followed for an average of 9.9 years. Number of deaths and average length of follow-up by race and genotype, stratified by baseline beta-agonist use, are presented in Table 4. Among black subjects using beta-agonists at baseline, 18% of those with Arg/Arg died during follow-up compared with 29% of those with Arg/Gly and 41% of subjects with Gly/Gly (25^th^ percentile survival time of>10 years for Arg/Arg, 9.0 years for Arg/Gly, and 5.5 years for Gly/Gly). This difference in crude mortality rates by genotype (Fig. 2) was not statistically significant (p = 0.33). Among white subjects reporting beta-agonist use at baseline, 22% of those with Arg/Arg died during follow-up compared to 25% with Arg/Gly and 27% with Gly/Gly (p = 0.27). We found no evidence of a difference in survival rates by genotype among blacks or whites who did not use beta-agonists. Controlling for age, sex, baseline FEV_1_/FVC ratio, baseline respiratory symptom score, and baseline smoking status, blacks using beta-agonists at baseline with the Arg/Gly genotype had better survival than black beta-agonist users with other genotypes [hazard ratio of 2.5 for Arg/Arg compared to Arg/Gly and 5.4 for Gly/Gly compared to Arg/Gly, p = 0.04]. Crude and adjusted hazard ratios by race and beta-agonist use are presented in Table 5.

**Table 4:** Number of deaths and average length of follow-up by race and genotype for subjects with (top) and without (bottom) baseline beta-agonist use

	Blacks with baseline beta-agonist use			Whites with baseline beta-agonist use		
	Arg/Arg	Arg/Gly	Gly/Gly	Arg/Arg	Arg/Gly	Gly/Gly
	n = 11	n = 35	n = 17	n = 27	n = 88	n = 74
Number (%) of deaths	2 (18%)	10 (29%)	7 (41%)	6 (22%)	22 (25%)	20 (27%)
Mean person-years of follow-up	9.3	9.1	8.2	9.4	9.3	9.1

	Blacks without baseline beta-agonist user			Whites without baseline beta-agonist user		
	Arg/Arg	Arg/Gly	Gly/Gly	Arg/Arg	Arg/Gly	Gly/Gly
	n = 960	n = 1942	n = 1051	n = 1422	n = 5116	n = 4191
Number (%) of deaths	119 (12%)	242 (13%)	142 (14%)	112 (8%)	354 (7%)	315 (8%)
Mean person-years of follow-up	9.7	9.7	9.6	10.0	10.1	10.1

**Table 5:** Crude and adjusted hazard ratios by race and baseline beta-agonist use

Variable	Estimated Crude Hazard Ratio (95%CI) *Estimated Adjusted Hazard Ratio (95% CI)* *			
	Blacks without β-agonist use	Whites without β-agonist use	Blacks with β-agonist use	Whites with β-agonist use
Age (10 yr increment)	2.5 (2.2, 3.0)	3.1 (2.7, 3.5)	6.3 (2.5, 16.0)	3.1 (1.7, 5.7)
	*2 .4 (2.0, 2.8)*	*2.9 (2.5, 3.3)*	*8.1 (2.3, 28.9)*	*2.5 (1.3, 5.1)*
Sex (0 = male,1 = female)	0.6 (0.5, 0.7)	0.5 (0.4, 0.6)	0.2 (0.1, 0.4)	0.8 (0.4, 1.4)
	*0.8 (0.7, 1.0)*	*0.6 (0.6, 0.7)*	*0.2 (0.1, 0.6)*	*1.0 (0.6, 1.9)*
FEV_1_/FVC ratio (1% increment)	1.0 (0.9, 1.0)	1.0 (0.9, 1.0)	1.0 (1.0, 1.0)	1.0 (0.9, 1.0)
	*1.0 (0.9, 1.1)*	*1.0 (0.9, 1.1)*	*1.0 (0.9, 1.1)*	*1.0 (0.9, 1.0)*
Log of respiratory symptom score	1.1 (1.1, 1.2)	1.1 (1.1, 1.2)	1.0 (0.8, 1.2)	0.9 (0.8, 1.1)
	*1.1 (1.1, 1.1)*	*1.1 (1.1, 1.1)*	*1.1 (1.0, 1.1)*	*0.8 (0.7, 1.0)*
Smoking status (1 = current, 2 = former, 3 = never)	0.6 (0.6, 0.7)	0.6 (0.5, 0.6)	0.4 (0.2, 0.8)	0.4 (0.3, 0.60
	*0.7 (0.6, 0.8)*	*0.7 (0.6, 0.7)*	*0.6 (0.3, 1.2)*	*0.4 (0.3, 0.7)*
*ADRB2* genotype				
Arg16Arg	1.0 (0.8, 1.2)	1.2 (0.9, 1.4)	0.6 (0.1, 2.8)	0.9 (0.4, 2.2)
	*1.0 (0.8, 1.2)*	*1.2 (0.9, 1.4)*	*2.5 (0.4, 14.7)*	*2.1 (0.8, 5.5)*
Arg16Gly	*Referent*	*Referent*	*Referent*	*Referent*
Gly16Gly	1.1 (1.1,1.1)	1.1 (0.9, 1.3)	1.7 (0.7, 4.5)	1.1 (0.6, 2.0)
	*1.1 (0.9, 1.3)*	*1.1 (1.0, 1.3)*	*5.4 (1.5,20.4) * †	*1.3 (0.7, 2.5)*

* Adjusted for variables shown in leftmost column of this table

† p<0.05 for test of trend across genotype

A similar pattern in crude survival rates was seen among black subjects reporting beta-agonist use at visit 2 (n = 69), with those with the Arg/Arg genotype having better crude survival (25^th^ percentile survival of greater than 10 years for Arg/Arg, 8.8 years for Arg/Gly, and 7.9 years for Gly/Gly, p = 0.24). After controlling for age, sex, FEV_1_/FVC ratio at visit 2, respiratory symptoms at visit 2, and smoking status at visit 2, those with Arg/Arg had better survival than black beta-agonist users with other genotypes (adjusted hazard ratio 8.0 for Arg/Gly compared to Arg/Arg and 13.8 for Gly/Gly compared to Arg/Arg, p = 0.02). This pattern is inconsistent with that seen among blacks with baseline beta-agonist use, among whom those with the Arg/Gly genotype appeared to have better adjusted survival.

Among white subjects reporting beta-agonist use at visit 2, 21% of those with Arg/Arg died during follow-up compared to 20% with Arg/Gly and 18% with Gly/Gly (p = 0.88). We observed no differences in survival by genotype among black or white subjects not reporting beta-agonist use at visit 2.

## Discussion

To our knowledge, this is the first large, population-based study to examine the association between *ADRB2* Arg16Gly polymorphism, beta-agonist use, lung function, and mortality. We found better lung function among black beta-agonist users with the Arg/Arg genotype compared with the Arg/Gly and Gly/Gly genotypes. These findings were not seen among blacks who did not use beta-agonists or among whites regardless of beta-agonist use.

Our finding of better lung function among black beta-agonist users with the Arg/Arg genotype is at odds with results from other studies that found regular beta-agonist use associated with reduced peak flow rates [12] and more frequent exacerbations in asthmatics with an Arg16 allele [28] as well as a recent randomized clinical trial [10] that found that regular beta-agonist use produced an improvement in lung function among Gly/Gly subjects but a deterioration in lung function among Arg/Arg subjects. Unfortunately, the number of beta-agonist users in our study is too small to permit analysis of temporal changes in lung function by genotype and race.

A possible explanation for the positive association we observed between Arg/Arg and pulmonary function among black beta-agonist users is that the Arg/Arg genotype is associated with less severe respiratory disease. Black beta-agonist users with the Arg/Arg genotype had lower median respiratory symptom scores and were less likely to have obstructive lung disease and to use inhaled corticosteroids compared to other genotypes. These features were similar across genotypes among white beta-agonist users. Thus, we cannot rule out the possibility that our findings are related to black beta-agonist users with the Arg/Arg genotype having less severe respiratory disease (and requiring less beta-agonist medication). Furthermore, both black subjects and those with the Arg/Arg genotype were more likely to be never smokers. Although we attempted to control for this difference by including baseline smoking status in the regression models, residual confounding by smoking status cannot be excluded.

Our findings regarding the association between *ADRB2* Arg16Gly polymorphism, beta-agonist use, and all-cause mortality are inconclusive. Data from the baseline clinical visit suggest black beta-agonist users with the Arg/Gly genotype have better survival, whereas data from the second clinical visit suggest that black beta-agonist users with the Arg/Arg genotype have better survival. This fluctuation may be due to the small numbers of black beta-agonist users with each genotype. However, the results are consistent in that both suggest that black beta-agonist users with an Arg16 allele may have lower all-cause mortality than Gly/Gly homozygotes, after controlling for important factors like age, smoking status, and respiratory symptom score. Our mortality findings highlight the need for additional studies of sufficient size and statistical power to allow examination of differential mortality among beta-agonist users of different races and genotypes.

This analysis has several limitations. Because statistical power is limited by the small number of black beta-agonist users (n = 62), we cannot rule out the possibility that our findings are due to chance. The small number of beta-agonist users precluded an analysis of temporal trends to examine if outcomes improved over time. Medication use was self reported and limited to the two-week period prior to each clinic visit. We had no data on haplotypes that have been described in relation to response to beta-agonists, such as haplotypes including the *ADRB2* Gln27Glu polymorphism [29]. Our results among black beta-agonist users may not be generalizable if the pattern of genetic variation in the black subsample of our study population differs in significant ways from other black populations.

In summary, we observed that black beta-agonist users with the Arg/Arg genotype at codon 16 of the *ADRB2* had better lung function, and, possibly, better overall survival compared to black beta-agonist users with the Gly/Gly genotype. Why we observe an association of the Arg/Arg genotype with outcomes among black but not white beta-agonist users is unclear and could be due to differential severity of respiratory disease, underlying differences in response to beta-agonist medication, or chance. Response to beta-agonists is undoubtedly a complex process influenced by many genes, and it is possible that associations seen with the Arg16Gly polymorphism are due to linkage disequilibrium with other polymorphisms whose prevalence differs by race (summarized in supplemental table S1). Additional studies with sufficient statistical power to detect differences between races will be critically important to improve understanding of this potentially important association.

## Supporting Information

Table S1 Measures of linkage disequilibrium between ADRB2 Arg16Gly (rs1042713) and Gln27Glu (rs1042714) polymorphisms by race/ethnicity
(0.05 MB DOC)

Text S1 Derivation of respiratory symptom score
(0.03 MB DOC)
