# Supplementary material for: ADRB2 Arg16Gly Polymorphism, Lung Function, and Mortality: Results from the Atherosclerosis Risk in Communities Study
Source: PLoS One. 2007 Mar 14;2(3):e289. doi: 10.1371/journal.pone.0000289 (PMC1808432; doi:10.1371/journal.pone.0000289)
Supplement: Table S1 — Measures of linkage disequilibrium between ADRB2 Arg16Gly (rs1042713) and Gln27Glu (rs1042714) polymorphisms by race/ethnicity (0.05 MB DOC) [file pone.0000289.s001.doc]

**Supplement: Table S1.** **Measures of linkage disequilibrium between *ADRB2* Arg16Gly (rs1042713) and Gln27Glu (rs1042714) polymorphisms by race/ethnicity**

| ***Reference*** | ***Sample*** | ***Measure of Linkage Disequilibrium*** | | | | |
| --- | --- | --- | --- | --- | --- | --- |
|  |  | **D** | **D’** | **r^2^** | **LOD** | **Levin’s δ** |
|  |  |  |  |  |  |  |
| HapMap [30] | US residents of Utah of northern and western European ancestry (n=90, 30 trios) (CEU) |  | 0.94 | 0.38 | 11.02 |  |
|  | Han Chinese in Beijing  (n=45 unrelated) (CHB) |  | 1.0 | 0.16 | 1.92 |  |
|  | Japanese in Tokyo (n=45 unrelated) (JPT) |  | 1.0 | 0.05 | 0.66 |  |
|  | Yoruba in Ibadan, Nigeria (n-90, 30 trios) (YRI) |  | 1.0 | 0.19 | 6.75 |  |
| Belfer  2005 [31] | US Caucasians (n=96 unrelated) |  |  | 0.32 |  |  |
|  | US African-Americans  (n=96 unrelated) |  |  | 0.19 |  |  |
| Hawkins 2006 [32] | US Whites (n=664)** |  | 0.99 |  |  |  |
|  | US African-Americans (n=521)** |  | 0.93 |  |  |  |
| Martinez  1997 [33] | US residents of Arizona of mixed Caucasian/Hispanic ethnicity (n=269)** |  |  |  |  | 0.48 |
| Dewar  1998 [34] | UK residents of Nottingham  (n=228 individual selected for atopy, bronchial hyperreactivity, or neither) | 0.38 |  |  |  |  |

* Two parents and one adult child comprise each trio.

** Subjects appear to be unrelated but this was not explicitly stated in the reference paper.
