# Supplementary material for: ADRB2 Arg16Gly Polymorphism, Lung Function, and Mortality: Results from the Atherosclerosis Risk in Communities Study
Source: PLoS One. 2007 Mar 14;2(3):e289. doi: 10.1371/journal.pone.0000289 (PMC1808432; doi:10.1371/journal.pone.0000289)
Supplement: Text S1 — Derivation of respiratory symptom score (0.03 MB DOC) [file pone.0000289.s002.doc]

**Supplement: Derivation of respiratory symptom score**

Subjects were assigned a respiratory symptom score ranging from 0 to 9 based on the number of affirmative responses to a set of 9 respiratory symptom questions, 4 of which asked about cough and 5 about wheeze. The respiratory symptom score was log-transformed for use in statistical analyses.

a.  Do you usually have a cough?
b.  Do you usually cough as much as 4 to 6 times a day, 4 or more days out of the week?
c.  Do you usually cough at all during the rest of the day [i.e. aside from first thing in the morning] or at night?
d.  Do you usually cough like this on most days for 3 consecutive months or more during the year?
e.  Does your chest ever sound wheezy or whistling apart from colds?
f.  Does your chest sound wheezy or whistling most day or nights?
g.  Have you ever had an attack of wheezing that has made you feel short of breath?
[If yes to *g.*], Have you had 2 or more such episodes?
[If yes to *g*.], Have you ever required medicine or treatment for the(se) attack(s)?
